# Incorporating American Sign Language Education to Prepare Future Physicians for Deaf and Hard-of-Hearing Patient Care

**DOI:** 10.1007/s40670-025-02600-8

**Published:** 2025-12-04

**Authors:** Sarah E. Hughes, May Rhee, Gabriella Auchus, Kate V. Panzer, Kelley Wyse, Heidi Joshi, Michael M. McKee

**Affiliations:** 1 University of Michigan Medical School, Ann Arbor, MI USA; 2 Department of Family Medicine, University of Michigan, Ann Arbor, MI USA; 3 Department of Family Medicine, University of Michigan Medical School, Ann Arbor, MI 48104 USA

**Keywords:** Deaf, Medical education, Deaf culture

## Abstract

A 10-week American Sign Language and Deaf health elective for medical students significantly improved knowledge of accessible communication strategies, disability laws, confidence arranging interpreters, and comfort with Deaf patients. This course demonstrates the impact of integrating targeted language and cultural competency training into medical curricula to advance equitable, accessible care.

Effective communication is fundamental to patient-centered care, yet Deaf and Hard-of-Hearing (DHH) individuals frequently encounter inaccessible healthcare environments [1]. An estimated 500,000 to 1 million Americans use American Sign Language (ASL) as their primary language, but many face delays in diagnosis, suboptimal treatment, and worse outcomes due to persistent communication barriers [2]. Despite federal mandates such as the Americans with Disabilities Act and Section 504 of the Rehabilitation Act, compliance remains inconsistent, and clinicians often lack awareness of these obligations or the skills to meet them [3]. Few medical schools offer structured training on how to care for DHH patients, leaving future physicians underprepared to meet the needs of this population [4]. Growing efforts across health professions programs have begun to integrate ASL and Deaf health education [4], reflecting increasing recognition of its importance while highlighting the need for broader implementation.

Since 2021, the University of Michigan Medical School has offered a 10-week ASL and Deaf health elective aimed at equipping students with foundational ASL skills, cultural competency, and practical communication strategies for improving accessibility in clinical settings. Designed for students with little or no prior ASL experience, the course integrates language instruction, along with a review of optimal communication strategies in clinical settings, Deaf culture and history, and disability law. Weekly 90-minute sessions primarily followed an “ASL-only” policy for language learning, with some activities (e.g., patient panels, simulations) conducted in spoken English, either by the presenter or through an ASL interpreter. This allowed for the course to integrate vocabulary and grammar relevant to healthcare encounters, discussions on Deaf cultural norms, strategies to improve communication accessibility, facilitate interpreter services and incorporate accessibility communication tools, and clarification of clinician and interpreter roles in healthcare with participation and assignments used for assessment.

Twenty-eight medical students (96% first-year) enrolled in the 2024 iteration, most identifying as female (64%), with 14% self-identifying as having a disability. Prior ASL experience was rare (82% beginners). Pre- and post-course surveys measured familiarity with DHH healthcare barriers, confidence in arranging ASL interpreters, knowledge of disability laws, comfort interacting with DHH patients, perceptions of healthcare accessibility, and preparedness to care for patients with disabilities. Responses used 4- and 5-point Likert scales. Descriptive statistics and paired t-tests evaluated change (Table 1).

**Table 1:** Pre- and post-course survey results

Measure	Pre-course mean (SD)	Post-course mean (SD)	*p*
Familiarity with DHH barriers^a^	2.14 (0.87)	3.09 (0.77)	< 0.001
Confidence in arranging interpreter services^a^	2.14 (0.87)	3.74 (0.71)	< 0.001
Knowledge of disability laws^a^	1.59 (0.74)	2.83 (0.69)	< 0.001
Comfort interacting with DHH patients^a^	3.28 (0.92)	3.96 (0.72)	< 0.001
Confidence in equitable care^a^	2.76 (0.88)	3.43 (0.75)	< 0.001
Perception of healthcare accessibility at institution^a^	2.52 (0.85)	3.39 (0.78)	< 0.001
Support for including ASL/Deaf health in curriculum^b^ (% Support)	55%	74%	< 0.001
Preparedness for caring for patients with disabilities^c^	1.59 (0.67)	2.96 (0.84)	< 0.001

^a^Rated on a 5-point Likert scale; 1 = not at all, 5 = extremely

^b^Rated on a 4-point Likert scale; 1 = useful, not a priority, 4 = essential, should be required; support = 3 or 4

^c^Rated on a 4-point Likert scale; 1 = not at all prepared, 4 = extremely prepared

Students’ confidence in arranging interpreters increased by 61.9%, familiarity with DHH healthcare barriers by 52%, and knowledge of disability laws by 59%, all statistically significant. Comfort interacting with DHH patients rose by 40%, and confidence in providing equitable care increased by 44.4%. Support for integrating ASL and Deaf health content into the medical curriculum rose from 55% to 74%.

Qualitative feedback reinforced the value of the course. Students consistently highlighted direct interaction with DHH community members as the most impactful element. One student noted, *“Meeting Deaf community members was the most meaningful part of the course—it made the experience feel real and impactful.”* Many requested more information on disability ethics, cochlear implantation, complex communication needs, and shadowing DHH patients to strengthen real-world skills.

Now in its fourth consecutive year, the elective continues to attract strong student interest; in the 2024 cohort, participation was associated with significant gains in knowledge, confidence, and preparedness to care for DHH patients. The integration of language acquisition, cultural competency, and healthcare accessibility training into a single elective offers a replicable model for other institutions. While these findings are based on self-reported data and limited to a single site, the consistent, statistically significant gains across all measured domains suggest broad applicability, though modest increase in absolute scores likely reflect the course’s brevity. Future directions include longitudinal follow-up to assess skill retention, expanded ethics content, objective proficiency assessments, and patient-reported outcome measures.

The persistence of communication inequities in healthcare underscores the urgency of incorporating Deaf health into medical education [4]. By embedding targeted ASL training alongside cultural and accessibility education, institutions can prepare future clinicians to meet legal obligations, foster patient trust, and address longstanding disparities. The sustained success with this elective illustrates that even brief, well-structured interventions can yield meaningful change in learner preparedness and awareness.
